# Real-World Impact of a Pharmacogenomics-Enriched Comprehensive Medication Management Program

**DOI:** 10.3390/jpm12030421

**Published:** 2022-03-08

**Authors:** Joseph P. Jarvis, Arul Prakasam Peter, Murray Keogh, Vince Baldasare, Gina M. Beanland, Zachary T. Wilkerson, Steven Kradel, Jeffrey A. Shaman

**Affiliations:** 1 Coriell Life Sciences, Philadelphia, PA 19112, USA; josephpjarvis@gmail.com (J.P.J.); aprakasam@coriell.com (A.P.P.); mkeogh@coriell.com (M.K.); vbaldasare@coriell.com (V.B.); skradel@coriell.com (S.K.); 2 Know Your Rx Coalition, University of Kentucky, Lexington, KY 40502, USA; gina.beanland@uky.edu (G.M.B.); z.wilkerson@uky.edu (Z.T.W.)

**Keywords:** pharmacogenomics, comprehensive medication management, cost analysis, clinical decision support systems, health resources, population health, personalized medicine, treatment outcome, adverse drug reactions, polypharmacy

## Abstract

The availability of clinical decision support systems (CDSS) and other methods for personalizing medicine now allows evaluation of their real-world impact on healthcare delivery. For example, addressing issues associated with polypharmacy in older patients using pharmacogenomics (PGx) and comprehensive medication management (CMM) is thought to hold great promise for meaningful improvements across the goals of the Quadruple Aim. However, few studies testing these tools at scale, using relevant system-wide metrics, and under real-world conditions, have been published to date. Here, we document a reduction of ~$7000 per patient in direct medical charges (a total of $37 million over 5288 enrollees compared to 22,357 non-enrolled) in Medicare Advantage patients (≥65 years) receiving benefits through a state retirement system over the first 32 months of a voluntary PGx-enriched CMM program. We also observe a positive shift in healthcare resource utilization (HRU) away from acute care services and toward more sustainable and cost-effective primary care options. Together with improvements in medication risk assessment, patient/provider communication via pharmacist-mediated medication action plans (MAP), and the sustained positive trends in HRU, we suggest these results validate the use of a CDSS to unify PGx and CMM to optimize care for this and similar patient populations.

## 1. Introduction

Pharmacogenomics (PGx) has been shown to play an important role in certain prescribing situations by identifying medication responders and non-responders to avoid specific adverse events and optimize drug dose [1,2]. Indeed, outcome-oriented studies repeatedly demonstrate positive impacts of PGx on individuals [3,4,5], providers [6,7], and the healthcare system [8,9]. Oncology [10,11] and psychiatry [12,13,14,15,16,17,18,19] implementations demonstrate particularly well-documented improvements. Further, a variety of positive economic, clinical, and humanistic outcomes (ECHO) associated with PGx have also been described. Thus, while implementational challenges exist [2,20,21,22], it is clear that PGx enhances healthcare delivery at multiple levels.

Likewise, comprehensive medication management is an established mechanism by which a patient’s medications (i.e., prescription, over-the-counter, supplements, etc.) are assessed by pharmacists to ensure that a given regimen is appropriate, safe, and effective. Many improvements in patient care have been observed by this approach to jointly consider medical history, conditions, comorbidities, medications, extrinsic factors, and desired outcomes [23,24]. Thus, like pharmacogenomics, comprehensive medication management (CMM) has also been shown to positively impact ECHO [25,26,27] despite similar implementation challenges of its own.

However, combining PGx testing and CMM services, empowered by a unifying clinical decision support system (CDSS) [28], promises to yield more valuable results by addressing individual patient needs in a more comprehensive fashion than either component can accomplish alone. This broader PGx-enriched comprehensive medication management (PGx + CMM) approach offers the potential to address many of the most pressing problems in prescription management including medication appropriateness, adherence, and adverse reactions in patients [29,30] through direct engagement of pharmacists who are uniquely well-trained and positioned to lead such efforts [31]. Additionally, this approach helps address practical limitations inherent in payor coverage models, an uncertain regulatory environment, practitioner education, the assessment of meaningful downstream clinical outcomes, as well as a lack of standardized testing and interpretation in a systematic way [32].

In 2017, the Teachers’ Retirement System of the State of Kentucky (TRS) partnered with Coriell Life Sciences (CLS) and the Know Your RX Coalition (KYRx) seeking to improve member health by reducing medication-related adverse events and increasing the success of medication therapies used by their Medicare Eligible Health Plan members. Consequently, CLS was engaged to offer TRS members the opportunity to participate in a PGx + CMM program that included member engagement and education, genetic testing, and pharmacist review by KYRx of broad medication risks.

**Figure 1:**
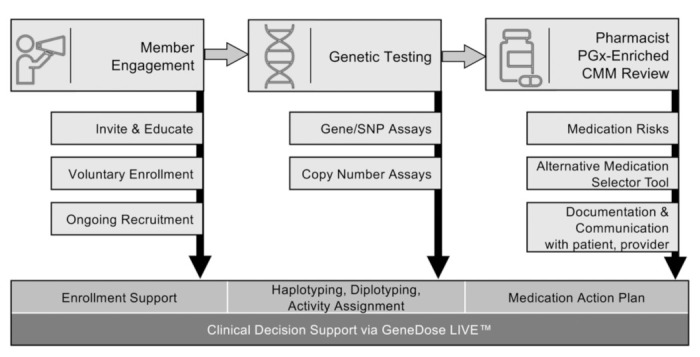
Program workflow for the comprehensive clinical decision support system (CDSS; GeneDose LIVE™, Coriell Life Sciences, Philadelphia, PA, USA) including pharmacogenomics-enriched comprehensive medication management (PGx + CMM). The CDSS enabled a wide variety of essential activities supporting the Quadruple Aim including member engagement and education, comprehensive pharmacogenetic testing, and pharmacist review. Moreover, the CDSS facilitated both the unification of PGx with CMM and the development of pharmacist-authored medication action plans (MAPs) that were observed to drive positive changes in economics and healthcare resource utilization. Abbreviations: PGx, pharmacogenomics; CMM, comprehensive medication management.

While long-promised, few large-scale, longitudinal datasets have been published evaluating the real-world impact of PGx and CMM on healthcare delivery. The particular PGx + CMM intervention implemented with TRS addressed practical limitations mentioned above, and also facilitated system-wide implementation by supporting a wide variety of activities (Figure 1) including participant enrollment, education, PGx testing, pharmacist training, medication appropriateness and risk determination, PGx-enriched comprehensive medication management review, alternative medication evaluation, tele-health interactions, ongoing participant outreach, and ultimately the development of a comprehensive medication action plan (MAP).

In this retrospective study of a real-world implementation, we document the direct outcomes of this program on direct medical costs and healthcare resource utilization in patients ≥65 years of age in the first 32 months after program initiation. We conclude with a discussion on the impacts in this population and further application in more diverse population samples.

## 2. Methods

### 2.1. Program Description

Members of the Teachers’ Retirement System of the State of Kentucky (TRS) were invited to enroll via a mailed announcement which included educational material tailored for an older adult population and a letter from TRS’s Executive Secretary explaining the goals and benefits of the program. TRS membership consists of, and is restricted to, Kentucky’s public school teachers who are retired and Medicare eligible, and their spouses. Enrollment in the PGx + CMM program consisted of a brief phone call, web-based survey, or in-person event to collect contact, medication, diet, and lifestyle (e.g., smoking and alcohol consumption) information. Additional mailings, phone outreach, webinars, an information website, and newsletters were also used as part of the ongoing recruitment process.

### 2.2. Genetic Testing

Upon enrollment, a DNA collection kit was sent to the participant for in-home saliva collection. The sample was then shipped to and analyzed by a CLIA- and CAP-licensed DNA sequencing laboratory (Quantigen Biosciences™, Fishers, IN, USA). The variants assayed were chosen based on documented clinical utility and their relationship to medication use outcomes (Table S1). Results were submitted via the GeneDose API (Application Programing Interface; Coriell Life Sciences, Philadelphia, PA, USA), a broadly interoperable service which received genotype data, demographic details, drug regimen lists, and other patient-specific information, which then initiated production of a clinical report. Genotype and Copy Number Variation (CNV) results were converted into diplotypes based on standard nomenclatures [33,34,35] and subsequently interpreted in the context of the patient-specific data collected at enrollment.

### 2.3. Medication Action Plan

Pharmacists (Know Your Rx Coalition, Lexington, KY, USA) utilized GeneDose LIVE™ (GDL; Coriell Life Sciences, Philadelphia, PA, USA), a comprehensive clinical decision support system (CDSS) to evaluate risks associated with a participant’s medication regimen. This evaluation involved detailed information on medications and indications, genetic and non-genetic sources of patient-specific risk, as well as mechanisms for the validation of regimen adjustments. The results were distilled by a clinical pharmacist to create a medication action plan (MAP) that summarized the proposed changes for the patient’s prescribing physician.

Specifically, genetic details included drug–gene level clinical recommendations and references to guidelines, drug labels, and primary literature from the CLS Knowledgebase—a curated collection of evidence-based literature. In practice, once clinical results were available, a program pharmacist initiated a telepharmacy consultation with the participant. The pharmacist then reviewed information collected during enrollment, confirmed the patient’s regimen, probed for patient goals and concerns (e.g., inadequate responses or side effects to current medications), and added any missing patient-specific information to the CDSS. A comprehensive medication risk assessment was then performed by analyzing the prepopulated CDSS Risk Chart—a data visualization of the magnitude of medication risks across nine potential sources. These distinct but not mutually exclusive risks include pharmacogenomics, drug–drug interactions (including phenoconversions), contraindications, anticholinergic burden, lifestyle factors (e.g., foods, alcohol, and smoking), pregnancy and lactation warnings, AGS Beers Criteria^®^, Food and Drug Administration (FDA) black box warnings, and pediatric-specific risks (see also Figure S1). Next, the pharmacist utilized the CDSS alternative medication selector interface to see a patient-specific risk analysis of each potential replacement medication for those they previously identified as requiring modification. This analysis allowed them to note medications that should be added, removed, modified (e.g., change dose), monitored (e.g., Adverse Drug Reactions (ADR), blood pressure), be alerted for potential future issues (i.e., pre-emptive warnings), or left unchanged. The pharmacist then selected medications that met the therapeutic intent with lower risk characteristics for that patient, from the available alternatives when appropriate. This process continued until a new regimen was developed with an improved medication safety and efficacy profile. Patient responses, information provided by the CDSS, and pharmacist clinical judgement all factored into pharmacist clinical practice and subsequent recommendations in the form of a patient-specific MAP. Proposed changes were discussed with the patient during their telepharmacy consultation and then communicated (e.g., faxed or emailed) to their healthcare providers. Rationales for recommending the change of any medication (e.g., genetic risk, drug interaction, and anticholinergic burden warning) were also captured to ensure effective communication with other healthcare professionals involved in the treatment plan.

### 2.4. Evaluation of the Program

In this retrospective study of a real-world implementation, deidentified administrative medical and pharmacy insurance data were used to establish baseline statistics and analyze changes over time in healthcare resource utilization and medical costs for members of TRS. Specifically, data for the 12 months pre-program period and for the first 32 months after program start were extracted and prepared for analysis. Continuously insured members—defined as those with a medical insurance claim in the 12 months prior to the start of the program, and at least one claim in the last six months of the program evaluation—aged 65 and greater were included in the economic analysis. Data from TRS members who did not enroll in the program functioned as a contemporaneous comparison cohort (i.e., “control group”) while TRS members who completed the program (i.e., had MAPs available for six months or greater) were explored as the “intervention group”. De-identified retrospective chart reviews of the medication risks and pharmacist recommended regimen changes were conducted for the intervention cohort to evaluate clinical impacts of the intervention.

This study was approved by the Biomedical Research Alliance of New York Institutional Review Board (BRANY; file number: 20-15-600-753, date of approval: 23 December 2020). While it was determined that the retrospective chart review did not constitute research involving human subjects, written consent for the use of the patient’s de-identified sample and information for research was nonetheless requested as part of the sample collection process and data from TRS members not consenting to research use were excluded from the retrospective chart review.

### 2.5. Economic Data Analysis

Invitations to participate in the PGx + CMM program (Figure 1) were sent to all active TRS members. Individuals with pharmacogenetic results and medication action plans (MAPs) available for more than six months were analyzed in order to ensure adequate time for claims adjudication; due to the inherent nature of the US healthcare claims documentation and approval process, any individual claim may not be complete until fully adjudicated. To explore program impacts at a system-wide level, de-identified insurance data for the intervention group were then compared to the members that did not participate. All de-identified MAPs for consented participants were analyzed.

Demographic information was captured from insurance claims to calculate basic statistics prior to program enrollment including age, sex, Charlson Comorbidity Index (CCI) [36], and number of medications. Statistical comparisons between the two groups were calculated utilizing Student’s t-test for difference in means, and chi-square test for difference in categorical frequencies. The medical claim costs in US dollars were aggregated by month for the intervention and the control groups to evaluate direct medical costs per member per month (PMPM). Potentially biasing high-cost claims associated with oncology and accidents (i.e., non-medication-related trauma and injuries) as determined by ICD-10 codes were excluded. The monthly PMPM difference for each group was then calculated and compared to the baseline mean difference for the 32 months following program start, using a difference-in-differences model. A difference-in-differences model is a quasi-experimental approach that estimates the difference in average outcome in the intervention group before and after program start compared to the difference in average outcome in the control group before and after program start. The estimated PMPM saved amount was calculated as the difference between the baseline difference and post-program difference. The cumulative cost savings for the intervention group is then estimated by multiplying the estimated PMPM saved amount, intervention group members, and 32 months of program duration.

A similar approach was utilized to calculate healthcare resource utilization (HRU) rates. Specifically, claims for each category of service (i.e., outpatient, inpatient, emergency) [37] were aggregated (Table S2) for each group at a monthly level. Rates were annualized and calculated per month, per 1000 members. The monthly HRU difference for the intervention group and control groups was then calculated and compared to the baseline mean difference for the 32 months following program start. Additionally, the intervention group’s estimated total claims avoided was calculated for the 32 month post-program period. The estimated claims avoided calculation is Intervention Baseline + (Control Post-Program—Control Baseline) * 32 month program duration. Welch’s t-tests were used to test the pre- and post-program baseline mean differences for direct medical costs, outpatient visits, emergency visits, and inpatient days. *p* values less than 0.05 were considered statistically significant.

## 3. Results

### 3.1. Retrospective Study: Intervention and Control Assignments

**Figure 2:**
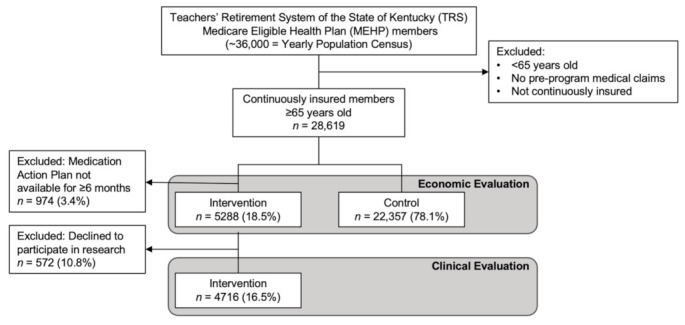
Study population definitions and inclusion/exclusion criteria. Invitations for the PGx + CMM program were sent to all active TRS members. Following minimal exclusions (age, continuous medical insurance coverage, and MAP availability), data from a total of 27,000 members were used in economic and clinical evaluations of program impact. Of these, 5288 individuals voluntarily participated in the program and were defined as the intervention group. Data from the remaining 22,357 individuals were used as a contemporaneous comparison (i.e., “control”) group in analyses of economic impact. The clinical evaluation included 4716 members of the intervention group who consented to be included in this research.

All active TRS members were invited to participate in the PGx + CMM program. Of those invited, 28,619 were determined to be continuously enrolled in their medical insurance plan (Figure 2). A total of 5288 completed their home DNA collection, participated in a consultation with a KYRx pharmacist, and thus had MAPs developed. The control group consisted of the 22,357 members that did not participate in the program. All de-identified MAPs for consented participants (*n* = 4716) were analyzed.

### 3.2. Demographic Data

At baseline—defined as the 12 calendar months preceding the program start—the characteristics of the intervention and control cohorts were similar. Specifically, the average age of the intervention and control cohorts was 73.7 versus 74.0, respectively, and the distribution of sex and Charlson Comorbidity Index (CCI) was 67% female and CCI = 3.6 in both cohorts (Table 1). The intervention group was on average prescribed one more medication than the control group (13.0 versus 11.9, *p* < 0.0001), with more participants prescribed 11 or more medications than the non-participant group. Additional member-specific demographic information was not available to this study, but publicly-accessible data suggest that fewer than 5% of the Kentucky teachers population are non-white [38] and describes that all certified teachers in the state possess at least a bachelor’s degree [39]. As such, analysis of this population sample is expected to be reasonably robust to impacts in unmeasured socioeconomic factors.

### 3.3. Medication Action Plan

The medication-related risks for each participant for whom a MAP was developed were assessed by the pharmacist performing the PGx + CMM review using the clinical decision support system (CDSS). The most prevalent risk in this population (*n* = 4716) was AGS Beers Criteria^®^ with 90% of participants prescribed a medication with the associated risk (Table 2). This was followed by drug–drug interactions (87%), FDA black box warnings (84%), genetics (66%), anticholinergic burden (56%), and lifestyle (e.g., food, drink, smoking; 25%). The CDSS captured pharmacists’ notes and recommendations for subsequent delivery to the participant and their prescribing physician. The most documented recommendation was “monitor”, with 78.8% of actionable MAPs alerting the physician to specific notes (i.e., ad hoc text entered by the pharmacist) to be alert to ADRs, efficacy of a medication, laboratory results (e.g., potassium levels), and biophysiological outcomes (e.g., blood pressure) (Table 3). The next-most documented category of recommendation was pre-emptive suggestions (22.1%) in cases where a new medication was already being considered or where there was a genetic concern associated with a commonly prescribed medication with known adverse reaction or efficacy issues. Recommended medication adjustments included discontinuation (15.6%), modifications (14.3%) with detailed instructions to change the dose, time of day, and/or taking a medication with or without food, and initiating a new medication (12.3%), and were attributed to specific genetic and non-genetic concerns in the pharmacists’ notes. MAPs were created for each member with genetic results, and all were conveyed to the member’s prescribing physician of record.

### 3.4. Economic Outcomes

**Figure 3:**
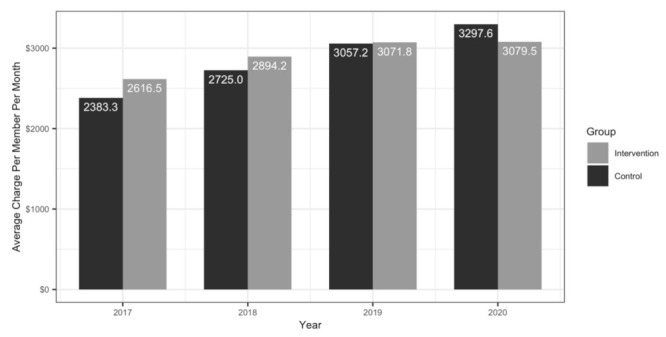
Yearly average charges to plan per patient per month (PMPM) for the identified intervention and control groups. In the 12 calendar months pre-program (2017), the intervention group (*n* = 5288) was associated with average medical insurance charges of $233.17 PMPM more than the control group (*n* = 22,357). During the first 32 months of the PGx + CMM program, the cost curve for the intervention group appears to flatten. In this same 32 month period, the intervention group showed a significant decrease of $218.34 PMPM compared to the control group, whose costs continued to increase linearly.

**Figure 4:**
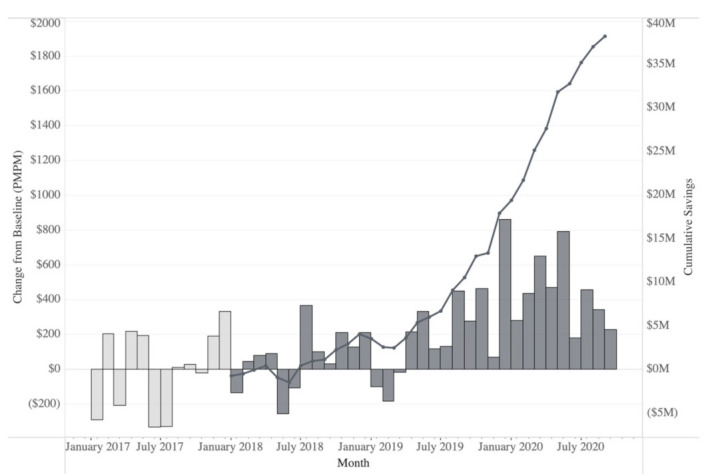
Savings charted as monthly differences (bars) from pre-program baseline and cumulative reduction (line) in direct medical charges in the identified intervention group compared to the identified control group calculated using direct medical charges from administrative medical insurance claims data. The baseline cost is calculated as the average per patient per month (PMPM) cost over the 12 months preceding the program implementation. During the baseline period, between January 2017 and January 2018 (i.e., prior to program implementation; light grey bars), monthly differences in cost fluctuate as expected. In the 32 months post-implementation, from January 2018 to August 2020, the intervention group shows consistent positive savings in direct medical charges (dark grey bars) PMPM, generating substantial cumulative savings (dark line). In total, the PGx + CMM program has achieved a reduction of approximately $7000 per patient in direct medical charges for a total of ~$37 million over the 5288 identified participants compared to the 22,357 identified non-participants—which calculates as an average savings of $218.34 PMPM.

A difference-in-differences model was used to quantify the change between the intervention and control groups, based on the shifts observed in the 32 months after the program initiation compared to the pre-program time period for each group. In the pre-program 12 months, the intervention group was associated with average charges of $233.17 PMPM more than the control group (Figure 3). However, during the first 32 months of the program, the intervention group showed a significant decrease of $218.34 PMPM (95% CI, −46.46–−391.01, *p* = 0.0151) compared to the control group, whose costs continued to increase linearly. The intervention group realized a calculated cumulative reduction in direct medical charges of $37,027,769 relative to the control during the first 32 months of the program (Figure 4). From a practical perspective, direct medical charges do not include the Medicare discounts and fee schedule, any co-insurance, deductibles, and any plan-specific negotiated rates but do provide a universal measure of cost that can be applied as a top line comparator to any health system.

Additionally, though cost reduction was not a goal of the program, we analyzed administrative pharmacy insurance claims to gauge the effect of the PGx + CMM program on pharmacy claims costs. Tracking the general upward trend of national healthcare costs, pharmacy insurance claims increased in both the intervention and control groups from the pre-program period through the first 32 months of the program. Specifically, the trends observed in the pre-program period continued into the post-program period for both groups (Figure S2). This suggests that factors that contribute to the pharmacy spend are beyond the scope of this PGx + CMM program.

### 3.5. Healthcare Resource Utilization

**Figure 5:**
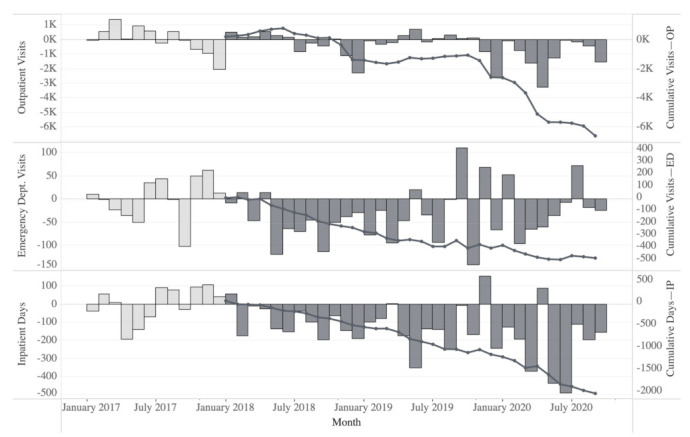
Monthly changes from baseline (average cost per patient per month over the 12 months preceding program implementation) and cumulative reduction in specific healthcare resource utilization (HRU) rates (per 1000 members) between the intervention and control groups. The impact of the PGx + CMM program on HRU was calculated using CMS place of service codes from administrative medical insurance claims data. The monthly HRU difference for the intervention group and control groups was calculated and compared to the baseline mean difference for the pre-program and 32 months following program start. The resulting differences are presented as the change from baseline for the pre-program (light grey bars) and intervention periods (dark grey bars) with cumulative amounts (dark line). The PGx + CMM program intervention group realized a consistent reduction in HRU compared to the control group in the evaluated outpatient (OP) visits, emergency department (ED) visits, and inpatient (IP) days.

A difference-in-differences analysis was also used to evaluate outpatient (OP) visits, emergency department (ED) visits, and inpatient (IP) days to explore the impact of the PGx + CMM program on the HRU, a proxy for patient health and wellness (Figure 5). At baseline, the intervention group had 31.98 fewer inpatient days (annual per 1000 members), 28.59 fewer ED visits, and 3085.59 more OP visits on average compared to the control group. During the first 32 months of the program, the intervention group had 173.01 fewer IP days on average compared to the control, a significant decrease of 141.03 days (95% CI, −64.40–217.65, *p* = 0.0008); 63.00 fewer ED visits on average, a decrease of 34.41 visits (95% CI, −69.62–0.79, *p* = 0.0549); 2664.95 more OP visits on average, a decrease of 420.64 visits (95% CI, −1074.33–233.06, *p* = 0.1945). We also compared healthcare utilization by type of service (see Table S2) during the pre- and post-program periods (Table 4).

## 4. Discussion

Here, we document multiple real-world economic and clinical outcome improvements, as measured by direct medical costs and healthcare resource utilization, produced by the deployment of a voluntary pharmacogenomics-enriched comprehensive medication management program in an older adult population. Comparing members that opted into the program to those who did not (i.e., identified interventions and controls, respectively), we observe an average calculated $218.34 PMPM savings. This equates to a cumulative $37 million savings in direct medical charges to the plan over the first 32 months post-implementation in the 5288 intervention members. In addition, we observed a 1.9%, 6.8%, and 14.9% reduction in outpatient, emergency department, and inpatient events, respectively, compared to expected healthcare resource utilization (HRU) (Table 4). Thus, we observe a positive shift in HRU away from acute services and toward more sustainable and cost-effective primary care options. Additionally, participating pharmacists anecdotally reported an increase in efficiency as well as an improved collaborative trust and communication with patients’ prescribing physicians. Ultimately, this program may generate improvements in each goal of the Quadruple Aim—lower costs, better outcomes, improved patient experience, and improved clinician experience.

One potential source of the large improvements observed in this analysis is the comprehensive nature of the intervention which combined best-of-breed components into a unified process of medication management. This program embraced the complexity and competing priorities inherent in healthcare delivery by bundling patient education and engagement, pharmacogenomic testing, comprehensive medication risk evaluation, pharmacist intervention, efficient clinical decision support tools, and dissemination of information to physicians.

Of particular note is the high quality of the pharmacogenomic information included in this intervention. As has been described elsewhere, it is the result of a rigorous selection process for the inclusion of genes, polymorphisms, and variants that have emerged from evidence-based evaluation using established criteria which is independent of the population at hand [1,33,40,41]. This process also helps ensure that “valuable” variants are assayed, thus creating a high breadth of coverage and ability to include new findings in the literature. As expected, 100% of participants had at least one variant known to impact medication therapy outcome and 66% of participants had genetic risks detected in a currently prescribed medication.

Another likely source of the strong trends we observed is the unique nature of the implementation that combined the science of PGx with the systematic evaluation of regimens provided by CMM, and empowered by a comprehensive CDSS. This provided the opportunity to examine outcomes at a high level, bridging the fractured nature of the healthcare system which includes generalists, specialists, outpatient, emergency care providers, and inpatient hospital facilities. By looking at administrative health insurance claims and measuring outcomes over the first 32 months of a combined PGx + CMM program, we were able to detect significant changes in economics and healthcare resource utilization. In addition, the program was designed with intrinsic scalability (i.e., the CDSS and MAP) and actioned by trained pharmacists communicating to program-naive prescribers to maximize potential benefits. There are few, if any, constraints on implementing this solution in other contexts as there is minimal disruption in the existing workflow of care delivery.

In addition to the effective engagement of participants, the unique structure of this program places pharmacists in a key role and can provide value even prior to a medication being prescribed. Such pre-emptive testing arises naturally through genome-informed approaches and represents a key enhancement in the CMM process. Moreover, PGx + CMM delivered by trained pharmacists creates an opportunity for these members of the healthcare community to use their training, knowledge, and experience in an especially strategic and impactful way. Furthermore, the time spent with the patient is highly impactful and imparts the potentially synergistic effects of improved adherence on outcomes and so savings. While recent literature highlights the challenges of implementing PGx or CMM programs, pharmacists are an under-utilized component of the healthcare delivery apparatus for integrating meaningful change into clinical practice. In environments where collaborative practice agreements exist, PGx + CMM bolsters the tools available to directly ensure more precise prescribing. Even here, where there was not a collaborative practice agreement, the plain and direct language of the pharmacist-created medication action plan (MAP) allowed for efficient communication of medication change recommendations.

The complexity of the healthcare delivery process and the science behind evidence-based decision-making requires the bundling of established PGx recommendations with the best practices in CMM, and the facilitation of patient and prescriber education. The CDSS serves this purpose in this implementation. This streamlining of several aspects, each of which can become rate-limiting under certain circumstances, makes it possible to efficiently deliver improved healthcare even in a disjointed healthcare system. Uniting the fragmented system’s tools, expertise, know-how, professional guidances, and data provides ample opportunity to increase system-wide confidence and scalability.

Finally, an oft-overlooked part of the story in healthcare implementations is the patient experience. In this unique real-world implementation, factors such as the confidence participants gained from close interaction with pharmacists, their understanding of the potential impact of genetics on their response to medications via educational outreach, the ability to complete the entire process without leaving home, as well as the security, privacy, and ease of the program appeared to be advantages. Indeed, moving the point of care to the patient’s home also has the potential to improve healthcare delivery for patients with substandard access to healthcare. These factors are especially important in an older adult population, for whom technology can be a barrier rather than a convenience.

Interestingly, while participant usage of prescription drugs is very well captured in the claims data, usage of over-the-counter (OTC) medications may also have been impacted by participation in the program. Several OTC medications also show some evidence of genetic factors influencing their safety and efficacy profiles. Thus, this implementation, through the use of the CDSS, provides the opportunity to improve the safety and efficacy of their usage. Common examples of such medications in this population include ibuprofen [42,43] and omeprazole [44]. So too, an evaluation of the outcomes of preemptive testing for OTCs in this population may expose additional benefits.

It was also notable that our intervention group was prescribed more medications on average than the control group. This suggests that those who could benefit from this intervention were more likely to enroll. A follow up study could provide additional insights into the outreach and education process and how it impacted participation.

In light of the disparate nature of healthcare delivery in a population such as TRS, which is unified by a common payor rather than a common provider, our approach was to implement a combination of best practices and to provide a unifying CDSS that is independent of the particular systems where patients receive their care. The CDSS thus became the critical tool to deliver precision medicine successfully at scale in similar scenarios, and the MAP became the output that drives outcome improvements.

However, while pharmacists delivered all of the MAPs to members’ prescribing physician of choice, we were unable to assess specific physician–patient interactions in our retrospective study design. Thus, the significant net effects observed at the level of systemwide billing cannot be attributed directly to any one of these unmeasured activities. Nevertheless, our real-world implementation of PGx + CMM demonstrated meaningful economic and clinical impacts. Ultimately, future studies focusing on the subtleties of program population, process, and delivery will be needed to investigate the specific causal factors that drive the observed outcomes.

As this ongoing PGx + CMM program continues, we expect to see continued downward bending of the cost curve—a sustained decrease in healthcare costs—and improved patterns of HRU for participants. This may even accelerate due the advancing age and healthcare burden of the participating members who were not prescribed any medications at the start of the program. In addition, these benefits are expected in any similar population dealing with issues of polypharmacy, and the more controlled usage of HRU ensures efficient use of healthcare dollars and other resources overall.

## 5. Conclusions

We document the impact of a large real-world implementation of a pharmacogenomics-enriched comprehensive medication management (PGx + CMM) program in the US. By deploying these tools, supported by a robust and efficient clinical decision support system, patients, healthcare providers, and the system as a whole benefited in synergistic ways. The observed reduced costs, meaningful shifts in the patterns of patient healthcare resource utilization, as well as other encouraging trends suggest that wide-spread adoption could significantly advance the goals of the Quadruple Aim in health systems globally.

## Figures and Tables

**Table 1:** Population characteristics for the intervention and control groups in the 12 month pre-program period.

Population Characteristics for Pre-Program Period			
Variables	Intervention (*n* = 5288)	Control (*n* = 22,357)	*p*-Value
Age in Years, avg (SD)	73.7 (5.7)	74.0 (6.2)	0.0007
65–74, *n* (%)	3249 (61%)	13,382 (60%)	<0.0001
75–84	1696 (32%)	6849 (31%)	
≥85	343 (6%)	2126 (10%)	
Sex, *n* (%)			
Female	3523 (67%)	14,893 (67%)	1
Male	1765 (33%)	7464 (33%)	
Charlson Comorbidity Index, avg (SD)	3.6 (1.4)	3.6 (1.5)	1
0–2, *n* (%)	1113 (21%)	4789 (21%)	0.3509
3–4	3144 (59%)	13,056 (58%)	
5+	1031 (19%)	4512 (20%)	
Number of Medications, avg (SD)	13.0 (8.4)	11.9 (8.3)	<0.0001
0, *n* (%)	79 (1%)	584 (3%)	<0.0001
1–2	223 (4%)	1281 (6%)	
3–4	429 (8%)	1997 (9%)	
5–7	786 (15%)	3788 (17%)	
8–10	904 (17%)	3703 (17%)	
11+	2867 (54%)	11,004 (49%)	

Abbreviations: avg, average; SD, standard deviation.

**Table 2:** Risks associated with pre-intervention regimens.

Risks Associated with Pre-Intervention Regimens	
Identified Risk	*n* (%)
AGS Beers Criteria^®^	4232 (90%)
Drug–Drug Interaction	4083 (87%)
ADR (FDA Black Box)	3978 (84%)
Genetic	3110 (66%)
Anticholinergic Burden	2645 (56%)
Lifestyle	1171 (25%)
Contraindication	9 (0%)
Total Interventions (MAPs)	4716 (100%)

Abbreviations: ADR, Adverse Drug Reactions; FDA, Food and Drug Administration.

**Table 3:** Pharmacist-suggested actions based on 3228 medication action plans with recommendations.

Pharmacist Recommendations (MAPs)	
Actionable MAPs *n* (%)	3228
Monitor *	2545 (78.8%)
Future Concern ^†^	714 (22.1%)
Discontinue Medication	502 (15.6%)
Modify Prescription	461 (14.3%)
Initiate New Medication	398 (12.3%)

* ADRs, efficacy, labs, physiology, etc. ^†^ Pre-emptive alert; medication not currently prescribed. Abbreviations: MAPs, Medication Action Plans.

**Table 4:** Healthcare resource utilization events avoided in the intervention group.

Healthcare Resource Utilization (Intervention Group)			
Place of Service	HRU Events		
	Expected *	Actual	% Avoided
Outpatient	315,058	309,126	1.9%
Emergency Department	7129	6644	6.8%
Inpatient	13,340	11,351	14.9%

* Calculated based on pre- and post-program differences between the intervention and control groups during the first 32 months of the program. The trends observed in the control group are used as a counterfactual to estimate the medical insurance claims in the intervention group had this group not received the intervention. The estimated value calculation is: Intervention Baseline + (Control Post-Program—Control Baseline) * 32 month program duration.

## Supplementary Materials

The following supporting information can be downloaded at: https://www.mdpi.com/article/10.3390/jpm12030421/s1, Figure S1: The comprehensive clinical decision support system (CDSS; GeneDose LIVE™, Coriell Life Sciences) Risk Chart—a data visualization of the magnitude of medication risks across nine potential sources; Figure S2: Average pharmacy claims cost per patient per month (PMPM) by year for the intervention and control groups; Table S1: Genes assayed for the PGx-enriched comprehensive medication management program; Table S2: Organization by the CMS place of service codes for professional claims for program evaluation. Reference [37] is cited in the supplementary materials.
