# Supplementary material for: Real-World Impact of a Pharmacogenomics-Enriched Comprehensive Medication Management Program
Source: J Pers Med. 2022 Mar 8;12(3):421. doi: 10.3390/jpm12030421 (PMC8949247; doi:10.3390/jpm12030421)
Supplement: Supplementary file 1 [file jpm-12-00421-s001.zip › jpm-1577816-supplementary.pdf]

Journal of
*Personalized
Medicine*

MDPI

Supplementary Materials

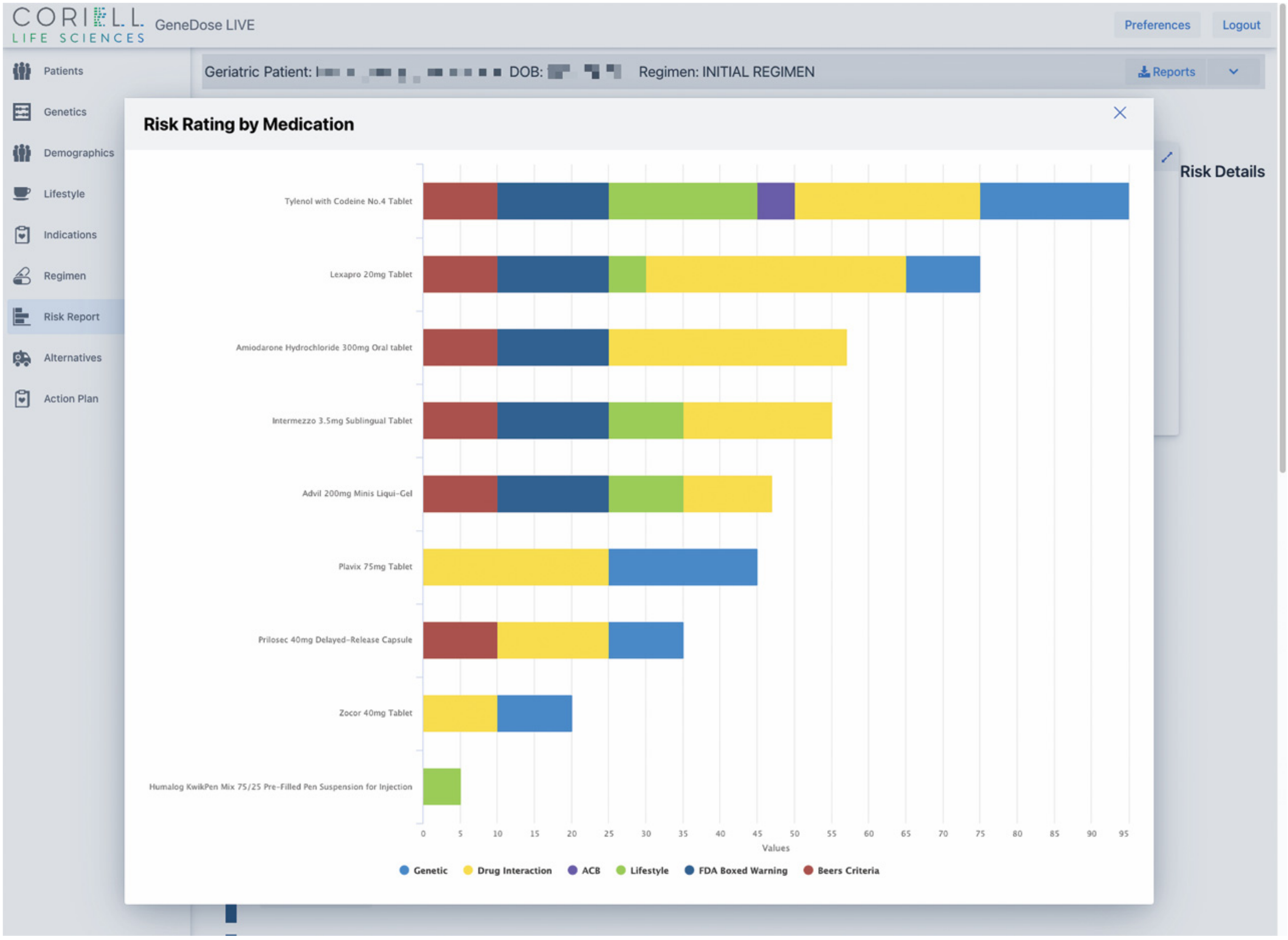

Figure S1. The comprehensive clinical decision support system (CDSS; GeneDose LIVE™, Coriell Life Sciences) Risk Chart—a data visualization of the magnitude of medication risks across nine potential sources. This CDSS facilitated both the unification of pharmacogenomics with comprehensive medication management (PGx + CMM) and the development of pharmacist-mediated medication action plans (MAPs) that were observed to drive large-scale changes in economics and healthcare resource utilization. Several additional CDSS tools are visible in the left-most menu bar.

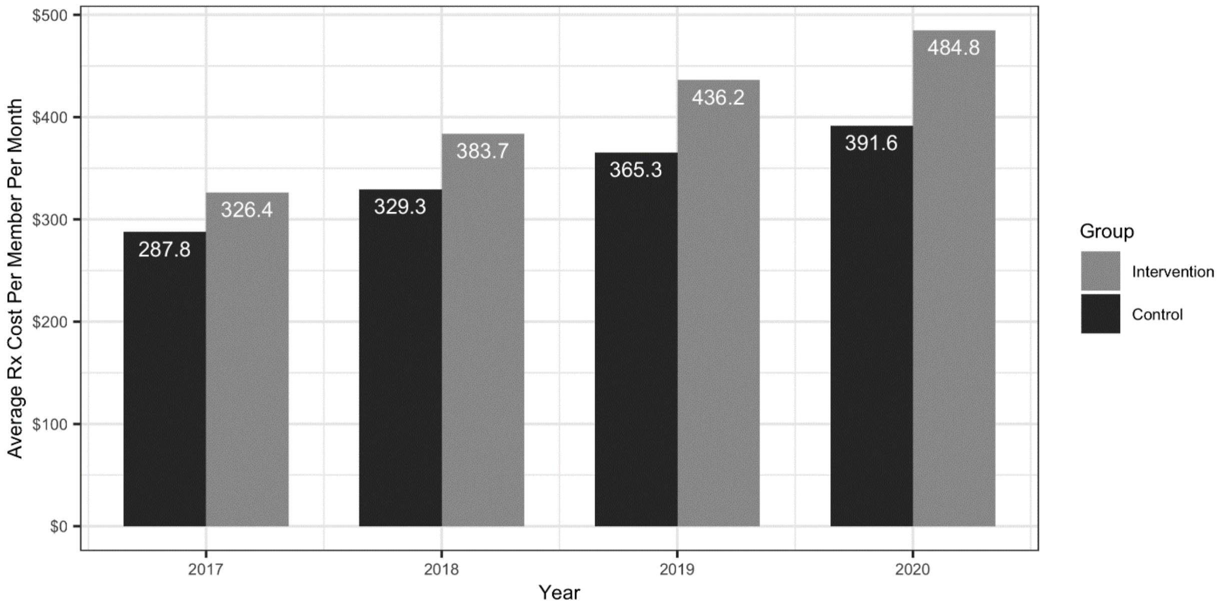

Figure S2. Average pharmacy claims cost per patient per month (PMPM) by year for the intervention and control groups. Abbreviation: Rx, prescription.

Table S1. Genes assayed for the PGx-enriched comprehensive medication management program.

Assayed Gene	Alleles	Reported
ADRA2A	c.-1291C>G	Yes
ANKK1	c.2137G>A	Yes
COMT	c.472G>A	Yes
CYP1A2	*1C, *1D, *1E, *1F, *1J, *1K, *1L, *1N, *1W	No
CYP2B6	*5, *6, *7, *16, *22, *34	No
CYP2C8	*1C, *2, *3, *4	Yes
CYP2C9	*2, *3, *4, *5, *6, *8, *11, *27	Yes
CYP2C19	*17, *4A, *4B, *5, *6, *7, *8, *9, *10, *3, *2	Yes
CYP2D6	*2A, *4A, *4J, *4M, *5, *6A, *6C, *7, *8, *9, *10A, *11A, *12, *14, *15, *17, *29, *35A, *36, *41, *64, *69, *82, *91, *109, *114, CNVs (duplications)	Yes
CYP3A4	*1B, *2, *3, *12, *17, *22	Yes
CYP3A5	*2, *3B, *3C, *6, *7, *8, *9	Yes
CYP4F2	*3	Yes
F2	c.*97G>A	Yes
F5	c.1601G>A	Yes
GRIK4	c.83-10039T>C	No
HLA-B	*1502	Yes
HTR2A	c.362-2211T>C	No
HTR2C	c.-759C>T	No
IFNL3	c.151-152G>A, g.1332A>C	No
MTHFR	c.1298A>C, c.677C>T	Yes
OPRM1	c.118A>G	Yes
SLCO1B1	*5	Yes
VKORC1	*2	Yes

A DNA collection kit (Oragene•Dx® OGD-510, DNA Genotek, Ontario, Canada) was sent to the enrollee for sample collection. Allelic discrimination was performed by TaqMan® OpenArray Genotyping on the QuantStudio™ 12K Flex Real-Time PCR System (Thermo Fisher Scientific, Waltham, MA, USA) according to standard protocols. Copy number variation (CNV) determination of *CYP2D6* exon 9 was evaluated on the same instrument utilizing CopyCaller® software. For each *CYP450* gene, *SLCO1B1*, *TPMT*, and *VKORC1* genes, the absence of a positive assay result for an included variant resulted in a *1 (wild type) designation.

Table S2. Organization by the *CMS Place of Service Codes for Professional Claims* for program Evaluation.

Category	Code	Place of Service	Code	Place of Service
Outpatient	3	School	49	Independent Clinic
	5	Indian Health Service	50	Federally Qualified Health Center
	11	Office	52	Psychiatric Facility-Partial Hospitalization
	15	Mobile Unit	53	Community Mental Health Center
	17	Walk-in Retail Health Clinic	57	Non-residential Substance Abuse Treatment Facility
	18	Place of Employment–Worksite	60	Mass Immunization Center
	19	Off Campus–Outpatient Hospital	62	Comprehensive Outpatient Rehabilitation Facility
	20	Urgent Care Facility	65	End-Stage Renal Disease Treatment Facility
	22	On Campus–Outpatient Hospital	71	Public Health Clinic
	24	Ambulatory Surgical Center	72	Rural Health Clinic
Inpatient	21	Inpatient Hospital		
	51	Inpatient Psychiatric Facility		
	61	Comprehensive Inpatient Rehabilitation Facility		
Emergency	23	Emergency Room–Hospital		
	41	Ambulance–Land		
	42	Ambulance–Air or Water		

List of available Place of Service codes [37] in the analyzed administrative medical claims and their categorization as outpatient, inpatient, or emergency.
